# Spatiotemporal gait patterns in individuals with unilateral transfemoral amputation: A hierarchical cluster analysis

**DOI:** 10.1371/journal.pone.0279593

**Published:** 2022-12-22

**Authors:** Daisuke Ichimura, Ryo Amma, Genki Hisano, Hiroto Murata, Hiroaki Hobara

**Affiliations:** 1 Artificial Intelligence Research Center, National Institute of Advanced Industrial Science and Technology (AIST), Tokyo, Japan; 2 Department of Mechanical Engineering, Tokyo University of Science, Chiba, Japan; 3 Department of Systems and Control Engineering, Tokyo Institute of Technology, Tokyo, Japan; 4 Research Fellow of the Japan Society for the Promotion of Science (JSPS), Japan; 5 Faculty of Advanced Engineering, Tokyo University of Science, Tokyo, Japan; University of Illinois at Urbana-Champaign, UNITED STATES

## Abstract

Gait pattern classification in individuals with lower-limb amputation could help in developing personalized prosthetic prescriptions and tailored gait rehabilitation. However, systematic classifications of gait patterns in this population have been scarcely explored. This study aimed to determine whether the gait patterns in individuals with unilateral transfemoral amputation (UTFA) can be clustered into homogeneous subgroups using spatiotemporal parameters across a range of walking speeds. We examined spatiotemporal gait parameters, including step length and cadence, in 25 individuals with UTFA (functional level K3 or K4, all non-vascular amputations) while they walked on a split-belt instrumented treadmill at eight speeds. Hierarchical cluster analysis (HCA) was used to identify clusters with homogeneous gait patterns based on the relationships between step length and cadence. Furthermore, after cluster formation, post-hoc analyses were performed to compare the spatiotemporal parameters and demographic data among the clusters. HCA identified three homogeneous gait pattern clusters, suggesting that individuals with UTFA have several gait patterns. Further, we found significant differences in the participants’ body height, sex ratio, and their prosthetic knee component among the clusters. Therefore, gait rehabilitation should be individualized based on body size and prosthetic prescription.

## Introduction

Although the prevalence of limb amputation is expected to reach 1 in 95 Americans by 2050 [1], less than 20% of those with lower-limb amputation can walk indoors and outdoors without walking aids, such as crutches, canes, or wheelers [2]. Walking is a fundamental component of any mobility strategy in daily live and could reduce the risk of obesity and depression in this population [3, 4]. Thus, increased understanding of locomotion in individuals with amputation is an indispensable prerequisite to improve gait rehabilitation and prosthetic technology, and help in clinical decision-making. Previous studies have reported on gait kinematics and kinetics to identify typical gait patterns in individuals with amputation such as the effect of socket configuration and alignment on gait [5], gait pattern changes with the level of amputation [6], and compensatory mechanisms of gait in terms of energy efficiency [7, 8]. However, systematic classifications of gait patterns to base clinical evaluation or criteria have scarcely been explored in individuals with lower-limb amputation.

Theoretically, walking speed is the product of cadence and step length. As both these parameters are inversely correlated at a given speed, an increase in one parameter will cause proportional decrease in the other. Prosthesis users with unilateral transtibial amputation have been shown to have greater variability in cadence and stride length than healthy participants across a range of speeds [9], indicating the existence of multiple gait patterns in prosthesis users. Gait pattern classification in individuals with lower-limb amputation may be useful for the appropriate evaluation of prostheses and targeted gait rehabilitation; however, little is known of gait patterns in individuals with lower-limb amputation, especially in those with unilateral transfemoral amputation (UTFA).

An effective method to subgroup homogeneous gait patterns in individuals with UTFA is the unsupervised machine learning technique, which is a method developed to identify basic patterns and relationships in datasets without labeled, classified, or categorized information [10, 11]. The technique is not a predetermined or arbitrary classification, but one based only on input data, which could ensure fairness [12, 13]. Hierarchical cluster analysis (HCA), a type of unsupervised machine learning, is widely used to classify gait patterns among patients with cerebral palsy [14, 15], Charcot-Marie-Tooth disease [13], and chronic stroke [16], as well as in healthy individuals [17–19]. Previous studies have shown that the physique and types of prosthetic knee joints affect gait function in individuals with UTFA [20]. Furthermore, gait biomechanics in individuals with UTFA could be affected by their age, physique, muscle strength, and the prosthesis they use.

Therefore, our primary objective was to investigate the presence or absence of several gait patterns among participants with UTFA by clustering their gait patterns into homogeneous subgroups using spatiotemporal parameters (e.g., cadence and step length) over a range of walking speeds. A secondary objective was to identify differences in demographic and spatiotemporal parameters among these subgroups. We hypothesized that (i) more than one cluster would be present in participants with UTFA and (ii) these clusters would differ according to participant demographic data.

## Methods

### Participants

Twenty-five participants with UTFA (18 male and 7 female participants; mean ± standard deviation age, 30.3 ± 9.0 years; body mass, 65.3 ± 14.3 kg; body height, 1.66 ± 0.75 m) participated in this study. All participants were accustomed to their habitual mechanical or microprocessor prosthetic knees and mechanical feet. The inclusion criteria required that participants had a Functional Classification Level of K3–K4 and were able to ambulate without using external aids or assistance. The exclusion criteria included neuromuscular disorders, functional lower-limb limitations, or health concerns that might affect standing or walking (e.g., low back pain). This study was approved by the appropriate Institutional Review Board (Environment and Safety Headquarters, Safety Management Division, National Institute of Advanced Industrial Science and Technology) and conducted following the guidelines set out in the Declaration of Helsinki and its later amendments. Written informed consent was obtained from all participants before the experiment.

### Experimental procedures

**Fig 1:**
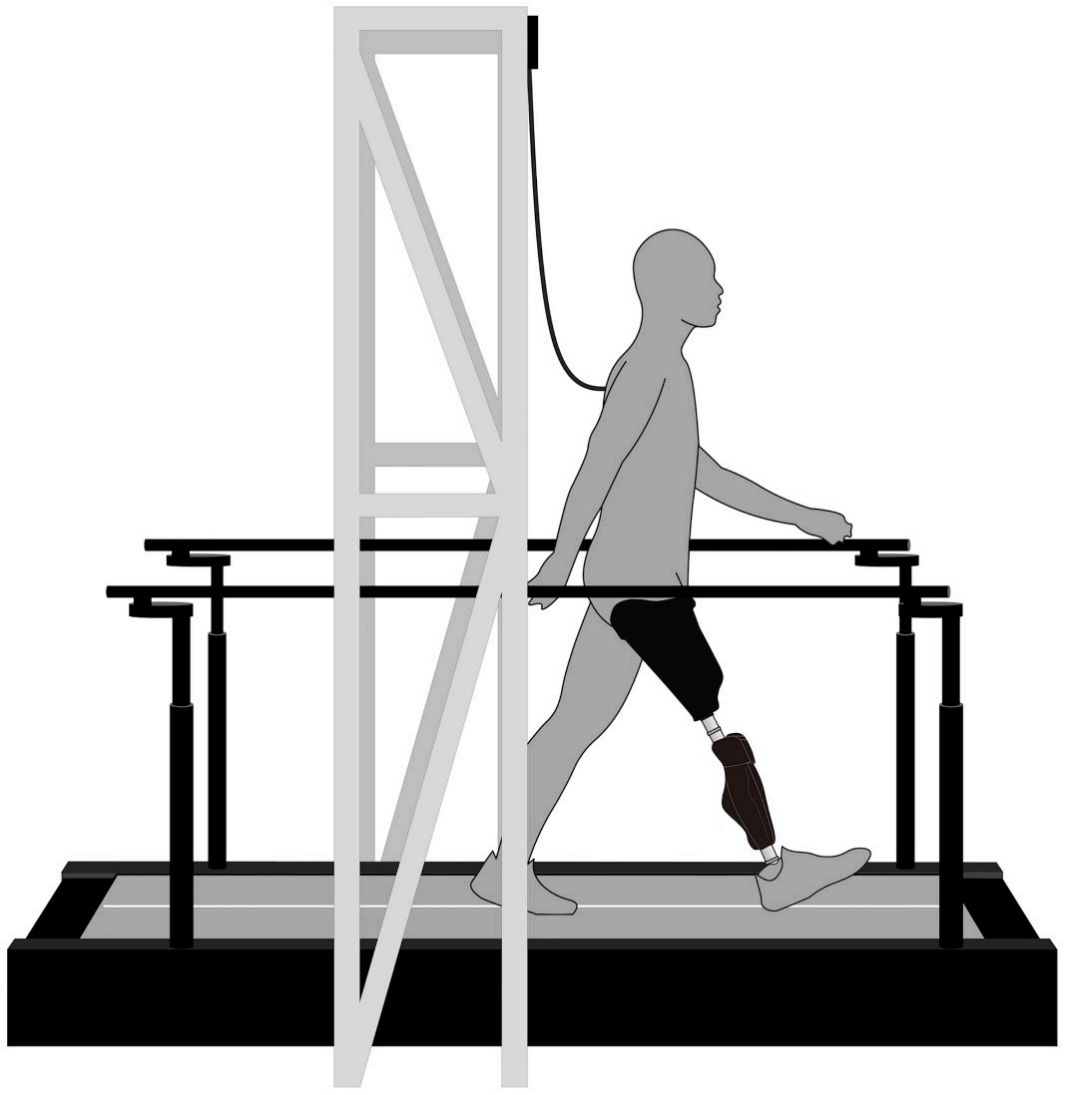
Schematic representation of the experimental setup. Participants with unilateral transfemoral amputation (UTFA) walked on a split-belt force-instrumented treadmill at eight speeds (2.0–5.5 km/h at increments of 0.5 km/h) for 30 s per speed category. The treadmill was equipped with a safety harness and two handrails to prevent falling.

As past findings showed that the differences in spatiotemporal parameters between treadmill and overground walking were negligible [21, 22], we used an instrumented treadmill for gait analysis in line with previous studies [23–25]. As in a previous study [23], all participants performed an adaptation trial for at least seven minutes to habituate to walking on the split-belt force-instrumented treadmill (Fig 1; FTMH-1244WA, Tec Gihan, Kyoto, Japan). During the habituation period, the participants experienced all experimental speeds (40–50 s for each), and we ensured that they could walk at each speed. The participants were then asked to walk for approximately 30 s at each of the eight speeds (2.0, 2.5, 3.0, 3.5, 4.0, 4.5, 5.0, and 5.5 km/h). No special instructions for spatiotemporal patterns were given to the participants. The treadmill was equipped with a safety harness to prevent falls. The safety harness was adjusted during all trials. We confirmed that the harness had adequate slack to avoid influencing natural walking. To minimize fatigue effects, we set adequate rest periods between the trials for all participants. Furthermore, we obtained introspection from each participant that the rest period was sufficient.

### Data collection and analysis

The vertical ground reaction force (vGRF) was recorded through two six-degrees-of-freedom piezoelectric force plates (TF-40120-CL and TF-40120-CR, Tec Gihan, Kyoto, Japan) embedded in the treadmill at a sampling frequency of 1000 Hz. The vGRF data were filtered using a fourth-order zero-lag lowpass Butterworth filter with a cutoff frequency of 20 Hz [23]. We determined the timing of foot-contact and toe-off for both limbs using a vGRF threshold of 40 N [23]. We chose valid steps using vGRF time series plots. A mean of 27 ± 8 consecutive steps was used for each limb at each walking speed. We calculated the cadence (steps/s) as the inverse of the time from foot-contact to the contralateral foot-contact for the intact and prosthetic limbs at each walking speed. The step length was calculated by dividing the walking speed by cadence. Consecutive cadence and step length values were averaged for each walking speed and each limb.

The Mahalanobis distance criterion [16] was used to eliminate outliers. HCA was then conducted to identify clusters with homogeneous gait patterns using the cadence and step length of both limbs at each walking speed (32 variables: 2 parameters × 2 limbs × 8 speeds). Squared Euclidean distance was chosen as the metric for this analysis, and Ward’s linkage method was adopted [10]. Individual clusters were continuously combined in the HCA to form new clusters. The procedure ended by grouping all trials into a single cluster that formed a hierarchical tree (a dendrogram). The final number of clusters was determined by the agglomeration coefficient while increasing the cluster number and adopting the “stopping rule” (a large percentage increase in coefficient reduction followed by a plateau) [10]. The number of clusters was also confirmed by the Mahalanobis distance criterion (>4.0) and visual inspection of the dendrogram [11, 26].

### Statistics analysis

After forming the clusters, one-way analysis of variance (ANOVA) was performed for each cadence and step length and demographic characteristic (age, body height, body mass, BMI, time since amputation, and current prosthesis use duration). The normality of the variables was tested using the Shapiro–Wilk test, and equality of variance with Levene’s test. When assumptions were not met, the non-parametric Kruskal–Wallis test was used. When a significant main effect was observed, post-hoc comparisons (*t*-test or Mann–Whitney *U* test) were performed, comparing the variables among the clusters. Other characteristics of the clusters (sex, etiology, residual limb length) were compared using Fisher’s exact test. Statistical significance was set to *P* < 0.05 and adjusted with Bonferroni’s correction. All statistical analyses were performed using RStudio (Version 1.1.456, RStudio, Inc.).

## Results

### General characteristics of the clusters

**Fig 2:**
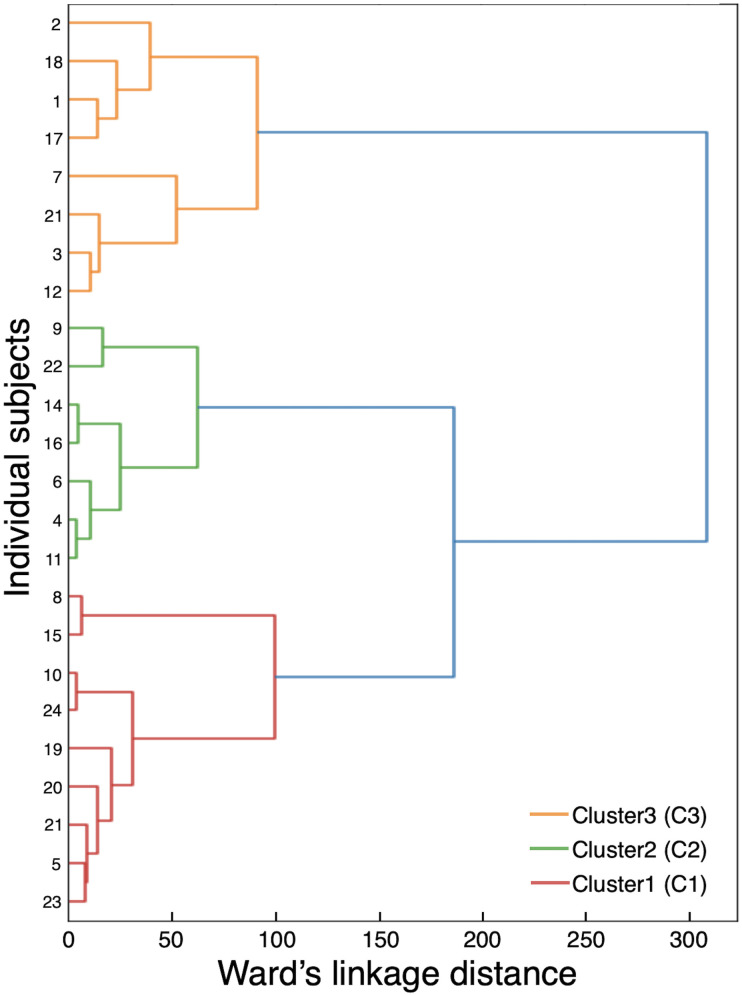
A dendrogram of the hierarchical cluster analysis. Linkage distance is shown on the horizontal axis, and the participants with unilateral transfemoral amputation (UTFA) are shown on the vertical axis. The three clusters are highlighted by red (Cluster 1, C1), green (Cluster 2, C2), and yellow (Cluster 3, C3).

After eliminating an outlier (Mahalanobis distance criterion > 4.0), a sample of 24 participants was used for further analyses. A large increase in the agglomeration coefficient reduction (87.8%) was noted between two and three clusters, followed by a plateau between three and four clusters (9.54% reduction). Therefore, we set the number of clusters to three. This result was confirmed by visual inspection of the dendrogram (Fig 2). Clusters 1 (C1), 2 (C2), and 3 (C3) comprised 9, 7, and 8 participants, respectively (Table 1).

**Table 1:** Demographic data of the three clusters (mean ± standard deviation).

	All subjects	Cluster 1	Cluster 2	Cluster 3	*P*-value
Sex					**0.023**
Male	17	9	3	5	
Female	7	0	4	3	
Age [years]	30.33 ± 9.97	30.00 ± 5.57	31.86 ± 10.57	29.36 ± 11.36	0.869
Body height [m]	1.65 ± 7.55	1.70 ± 0.04^b^	1.60 ± 0.07^a^	1.65 ± 0.08	**0.020**
Body mass [kg]	65.27 ± 14.30	72.45 ± 15.36	58.56 ± 10.97	63.07 ± 13.50	0.066
BMI [kg/m^2^]	23.63 ± 3.61	25.21 ± 4.32	22.78 ± 2.53	22.84 ± 3.31	0.376
Etiology					0.191
Trauma	14 (58.3%)	7 (77.8%)	5 (71.4%)	2 (25.0%)	
Sarcoma	6 (25.0%)	2 (22.2%)	1 (14.3%)	3 (37.5%)	
Cancer	2 (8.3%)	0 (0.0%)	0 (0.0%)	2 (25.0%)	
Congenital	2 (8.3%)	0 (0.0%)	1 (14.3%)	1 (12.5%)	
Time since amputation [years]	11.14 ± 8.18	10.79 ± 8.08	11.04 ± 10.74	11.64 ± 6.76	0.842
Prosthesis use duration [years]	2.67 ± 1.75	2.38 ± 1.88	2.01 ± 1.25	3.56 ± 1.80	0.196
Residual limb length					0.079
Short	4 (16.7%)	1 (11.1%)	2 (28.6%)	1 (12.5%)	
Middle	9 (37.5%)	2 (22.2%)	3 (42.9%)	4 (50.0%)	
Long	8 (33.3%)	6 (66.7%)	1 (14.3%)	1 (12.5%)	
Knee disarticulation	3 (12.5%)	0 (0.0%)	1 (14.3%)	2 (25.0%)	
Types of prosthetic knees					
NMPK	17	5	7	5	
MPK	7	4	0	3	
Percent with MPK	29.2%	44.4%	0.0%	37.5%	

Note that the definitions of residual limb length are as follows: “Short” less than 1/3 of the intact femur length, “Medium” less than 2/3 (and longer than 1/3) of the intact femur length, and “Long” longer than 2/3 of the intact femur length (but not knee disarticulation).

^a^ Significant differences from Cluster 1.

^b^ Significant differences from Cluster 2.

Abbreviations: BMI, body mass index; NMPK, non-microprocessor knee; MPK, microprocessor knee.

### Comparisons of demographic characteristics among clusters

The cluster characteristic data are represented in Table 1. C1 consisted of male participants only, while C2 and C3 comprised male and female participants (Table 1; *P* = 0.023). Furthermore, the body height in C1 was significantly greater than that in C2 (*P* = 0.020). Although it did not reach statistical significance (*P* = 0.066), the body mass in C1 was numerically the greatest, followed by that in C3 and C2. The percentage of microprocessor knees was 44.4% and 37.5% for C1 and C3, respectively, whereas C2 used only mechanical knee joints (Table 1). The groups were similar in age, body mass, etiology, time since amputation, current prosthesis use duration, and residual limb length among the three clusters (Table 1).

### Comparisons of spatiotemporal parameters among clusters

As shown in Tables 2 and 3, the cadence in C2 was significantly higher than that in C1 over a wide range of walking speeds in the intact limb and at 2.5 and 3.5 km/h in the prosthetic limb. The cadence in C2 was also significantly higher than that in C3 at 2.5 and 3.0 km/h in the intact limb (Table 2). However, the cadence was similar at all walking speeds for the prosthetic limb in C2 and C3 and for both limbs in C1 and C3 (Table 3).

**Table 2:** Spatiotemporal gait parameters of the intact limb (mean ± standard deviation).

Parameter	Speed	Cluster 1	Cluster 2	Cluster 3	*P*-value
Cadence [step/s]	2.0 [km/h]	1.46 ± 0.08	1.57 ± 0.06	1.43 ± 0.17	0.072
	2.5 [km/h]	1.55 ± 0.07^b^	1.73 ± 0.04^a^^,^^c^	1.61 ± 0.10^b^	**<0.001**
	3.0 [km/h]	1.68 ± 0.03^b^	1.84 ± 0.03^a^^,^^c^	1.74 ± 0.10^b^	**0.002**
	3.5 [km/h]	1.81 ± 0.07^b^	1.97 ± 0.04^a^	1.90 ± 0.11	**0.002**
	4.0 [km/h]	1.91 ± 0.06^b^	2.07 ± 0.08^a^	1.98 ± 0.13	**0.011**
	4.5 [km/h]	1.99 ± 0.05^b^	2.13 ± 0.10^a^	2.08 ± 0.15	**0.043**
	5.0 [km/h]	2.08 ± 0.07^b^	2.24 ± 0.12^a^	2.15 ± 0.14	**0.036**
	5.5 [km/h]	2.17 ± 0.06^b^	2.32 ± 0.12^a^	2.27 ± 0.13	**0.022**
Step length [m]	2.0 [km/h]	0.38 ± 0.02	0.36 ± (0.01)	0.36 ± 0.05	0.058
	2.5 [km/h]	0.45 ± 0.02^b^	0.40 ± (0.01)^a^^,^^c^	0.44 ± 0.03^b^	**0.001**
	3.0 [km/h]	0.50 ± 0.01^b^	0.45 ± 0.01^a^	0.48 ± 0.03	**0.002**
	3.5 [km/h]	0.54 ± 0.02^b^	0.50 ± 0.01^a^	0.52 ± 0.03	**0.003**
	4.0 [km/h]	0.58 ± 0.02^b^	0.54 ± 0.02^a^	0.56 ± 0.04	**0.012**
	4.5 [km/h]	0.63 ± 0.02^b^^,^^c^	0.59 ± 0.03^a^	0.60 ± 0.05^a^	**0.006**
	5.0 [km/h]	0.67 ± 0.02^b^	0.62 ± 0.03^a^	0.65 ± 0.05	**0.045**
	5.5 [km/h]	0.71 ± 0.02^b^	0.66 ± 0.04^a^	0.68 ± 0.04	**0.024**

^a^ Significant differences from Cluster 1.

^b^ Significant differences from Cluster 2.

^c^ Significant differences from Cluster 3.

**Table 3:** Spatiotemporal gait parameters of the prosthetic limb (mean± standard deviation).

Parameter	Speed	Cluster 1	Cluster 2	Cluster 3	*P*-value
Cadence [step/s]	2.0 [km/h]	1.34 ± 0.10	1.47 ± 0.12	1.34 ± 0.17	0.241
	2.5 [km/h]	1.44 ± 0.09^b^	1.60 ± 0.09^a^	1.49 ± 0.14	**0.014**
	3.0 [km/h]	1.55 ± 0.11	1.67 ± 0.06	1.59 ± 0.12	0.076
	3.5 [km/h]	1.65 ± 0.10^b^	1.79 ± 0.07^a^	1.71 ± 0.09	**0.020**
	4.0 [km/h]	1.75 ± 0.14	1.86 ± 0.08	1.81 ± 0.08	0.125
	4.5 [km/h]	1.81 ± 0.13	1.92 ± 0.09	1.89 ± 0.05	0.139
	5.0 [km/h]	1.9 ± 0.14	2.02 ± 0.11	1.96 ± 0.07	0.111
	5.5 [km/h]	1.97 ± 0.17	2.10 ± 0.13	2.05 ± 0.08	0.164
Step length [m]	2.0 [km/h]	0.42 ± 0.03	0.38 ± 0.03	0.42 ± 0.05	0.133
	2.5 [km/h]	0.48 ± 0.03^b^	0.44 ± 0.02^a^	0.47 ± 0.04	**0.021**
	3.0 [km/h]	0.54 ± 0.04	0.50 ± 0.02	0.52 ± 0.04	0.070
	3.5 [km/h]	0.59 ± 0.04^b^	0.54 ± 0.02^a^	0.57 ± 0.03	**0.021**
	4.0 [km/h]	0.64 ± 0.05	0.60 ± 0.02	0.61 ± 0.03	0.116
	4.5 [km/h]	0.69 ± 0.05	0.65 ± 0.03	0.66 ± 0.02	0.129
	5.0 [km/h]	0.74 ± 0.06	0.70 ± 0.04	0.71 ± 0.03	0.120
	5.5 [km/h]	0.78 ± 0.07	0.73 ± 0.05	0.74 ± 0.03	0.150

^a^ Significant differences from Cluster 1.

^b^ Significant differences from Cluster 2.

Tables 2 and 3 also show that C1 had significantly longer steps than C2 over a wide range of walking speeds in the intact limb and at 2.5 and 3.5 km/h in the prosthetic limb. The step lengths were similar at all walking speeds, except at 4.5 km/h in C1 and C3 and at 2.5 km/h in C2 and C3 in the intact limb (Table 2).

Overall, C2 was characterized by a higher cadence than the other two clusters over a wide range of walking speeds in both limbs. C1 was characterized by longer steps than the other clusters. Tables 2 and 3 show that the cadences and step lengths in C3 were nearly midway between those in C1 and C2.

## Discussion

The primary objective of this study was to investigate if walking patterns in individuals with UTFA could be clustered into homogeneous subgroups using spatiotemporal parameters across a range of walking speeds. The HCA identified three distinct and homogeneous walking gait patterns based on cadence and step length (Fig 1). To the best of our knowledge, this study is the first to demonstrate gait pattern classification in individuals with UTFA. The present study results support our initial hypothesis that more than one cluster would be present in individuals with UTFA. Furthermore, our results are consistent with those of previous reports that classified gait patterns into 2–5 clusters in patients with stroke [16], cerebral palsy [14], or neurological disorders [13]. The present study suggests that several gait patterns exist in individuals with UTFA.

The second hypothesis that these clusters would be separated based on demographic data was partially supported. It is especially worth noting that C1 consisted only of male participants who were taller and heavier than the participants in C2 and C3 (Table 1). Indeed, a previous study demonstrated that smaller height and body mass were potential risks of prosthetic knee buckling during walking in individuals with UTFA [20]. Furthermore, C1 steps in both limbs were longer than C2 and C3 steps (Tables 2 and 3), possibly because a larger body size tends to possess a larger muscle volume [27] with longer leg length. Additionally, a previous study found that the walking pattern is related to the residual limb length in unilateral transfemoral amputees [28]. In the present study, although it did not reach statistical significance, C1 included participants with a relatively longer residual limb (Table 1). Since the body size could be one of the factors affecting the spatiotemporal gait pattern in individuals with UTFA, it must be carefully considered during gait rehabilitation program assessment.

As shown in Tables 2 and 3, C2 was characterized by greater cadence than the other clusters in both limbs. Furthermore, the individuals in C2 were shorter in body height and lighter in body mass than those in the other subgroups (Table 1). Notably, 44.4% and 37.5% of those in C1 and C3, respectively, used microprocessor knees in their prosthetic limbs, while all those in C2 used mechanical prosthetic knees (Table 1). These results partially contrast past findings that the cadence achieved during walking with microprocessor knees was greater than that with mechanical knees at relatively slow walking speeds (2.4–3.1 km/h) [29, 30]. Our current findings suggest that demographic data, especially the prosthetic knee type and body size, could affect the spatiotemporal gait pattern in individuals with UTFA. Therefore, the combined effect of the prosthetic knee type and body size requires further investigation to provide better rehabilitation designs and prosthetic prescriptions to suit the needs of individuals with UTFAs.

Our results indicate that body size could affect the spatiotemporal gait patterns in individuals with UTFA, which is consistent with a previous study [31]. In particular, there is a linear relationship between stride length and cadence in healthy individuals, depending on walking speed [9]. This linear relationship, however, is disrupted in individuals with lower-limb amputation, implying the existence of more various gait patterns than those in healthy individuals. The present study also suggests the existence of multiple gait patterns in individuals with UTFA. Therefore, variations of gait patterns caused by differences in body size may exist greater in individuals with UTFA than in healthy individuals.

There are several concerns regarding the interpretation of our findings. First, we conducted HCA using cadence and step length in both limbs over a wide range of walking speeds. However, analysis of other features such as the center of mass or joint kinematics/kinetics might result in different clusters. In addition, such parameters, which widely differ in scale, may require normalization [32]. Therefore, future studies should investigate the effect of the input variables on gait pattern classifications in individuals with UTFA. Second, we recruited relatively young (30.3 ± 9.0 years) individuals with UTFA at functional level K3 or K4. However, spatiotemporal gait parameters vary with age [33] and the K level [34]. Further research is needed to confirm gait changes at different activity levels, including lower K levels. Third, as we recruited 18 male and 7 female participants with UTFA, the sex ratio of the sample population was uneven. Further research adopting a larger and more homogeneous group is needed to ensure that the results are more generalizable. Finally, although we compared demographic data among the three clusters, other factors might explain the difference in gait patterns. For example, gait mechanics are known to be influenced by several factors such as the very slow walking speeds [35], the socket fitting quality [36], the suspension system [37], alignment [38], and psychological factors [39]. Thus, these findings must be interpreted and generalized cautiously.

## Conclusion

The HCA used in this study identified three distinct and homogeneous walking gait patterns based on cadence and step length in individuals with UTFA. The gait patterns could be explained by the participants’ body sizes and the prosthetic knee components. Our results suggest that several gait patterns exist in individuals with UTFA and that gait rehabilitation in these participants should be individualized based on their body size and prosthetic prescription.

## Supporting information

S1 File (CSV)
